# Structural Connectivity is Differently Altered in Dementia with Lewy Body and Alzheimer’s Disease

**DOI:** 10.3389/fnagi.2015.00208

**Published:** 2015-11-02

**Authors:** Stefano Delli Pizzi, Raffaella Franciotti, John-Paul Taylor, Roberto Esposito, Armando Tartaro, Astrid Thomas, Marco Onofrj, Laura Bonanni

**Affiliations:** ^1^Department of Neuroscience, Imaging and Clinical Sciences, Aging Research Centre (CeSI), G. d’Annunzio University, Chieti, Italy; ^2^Department of Neuroscience, Imaging and Clinical Sciences, Institute for Advanced Biomedical Technologies (ITAB), G. d’Annunzio University, Chieti, Italy; ^3^Institute for Ageing and Health, Newcastle University, Newcastle upon Tyne, UK

**Keywords:** Alzheimer’s disease, dementia with Lewy bodies, diffusion tensor imaging, magnetic resonance imaging, structural connectivity

## Abstract

The structural connectivity within cortical areas and between cortical and subcortical structures was investigated in dementia with Lewy bodies (DLB) and Alzheimer’s disease (AD). We hypothesized that white matter (WM) tracts, which are linked to visual, attentional, and mnemonic functions, would be differentially and selectively affected in DLB as compared to AD and age-matched control subjects. Structural tensor imaging and diffusion tensor imaging (DTI) were performed on 14 DLB patients, 14 AD patients, and 15 controls. DTI metrics related to WM damage were assessed within tracts reconstructed by FreeSurfer’s TRActs Constrained by UnderLying Anatomy pipeline. Correlation analysis between WM and gray matter (GM) metrics was performed to assess whether the structural connectivity alteration in AD and DLB could be secondary to GM neuronal loss or a consequence of direct WM injury. Anterior thalamic radiation (ATR) and cingulum-cingulate gyrus were altered in DLB, whereas cingulum-angular bundle (CAB) was disrupted in AD. In DLB patients, secondary axonal degeneration within ATR was found in relation to microstructural damage within medio-dorsal thalamus, whereas axonal degeneration within CAB was related to precuneus thinning. WM alteration within the uncinate fasciculus was present in both groups of patients and was related to frontal and to temporal thinning in DLB and AD, respectively. We found structural connectivity alterations within fronto-thalamic and fronto-parietal (precuneus) network in DLB whereas, in contrast, disruption of structural connectivity of mnemonic pathways was present in AD. Furthermore, the high correlation between GM and WM metrics suggests that the structural connectivity alteration in DLB could be linked to GM neuronal loss rather than by direct WM injury. Thus, this finding supports the key role of cortical and subcortical atrophy in DLB.

## Introduction

Dementia with Lewy bodies (DLB) is the second most common form of neurodegenerative dementia after Alzheimer’s disease (AD) (Vann Jones and O’Brien, 2014).

Clinically, DLB patients present with greater attentional and visuo-perceptual impairment (Calderon et al., 2001; Collerton et al., 2003) and a less prominent memory loss (Collerton et al., 2003; Ferman et al., 2006) as compared with AD patients.

Recent studies on DLB patients have reported structural and functional connectivity alteration between cortical areas (Kantarci et al., 2010; Galvin et al., 2011; Kenny et al., 2012; Watson et al., 2012; Franciotti et al., 2013) and between cortex and subcortical structures (Kenny et al., 2013; Delli Pizzi et al., 2014a,b; 2015; Peraza et al., 2014).

In particular, Diffusion Tensor Imaging (DTI) has allowed the investigation of structural connectivity between brain areas by mapping the motion of water along neural axons and providing microstructural details about the shape and integrity of white matter (WM) fibers. Commonly used DTI metrics include fractional anisotropy (FA), mean diffusivity (MD), radial diffusivity (RD), and axial diffusivity (DA). FA and MD are associated with the primary degeneration of axons; FA is higher in organized than in disorganized fascicles, which are affected by microstructural processes such as demyelination, axonal degradation, or gliosis (Pierpaoli et al., 1996). MD, in contrast, is a sensitive, albeit rather non-specific, measure that can be increased by any pathological process affecting the cell membranes (Bosch et al., 2012). RD provides more detailed information about breakdown of myelin (Song et al., 2002, 2003), whereas DA describes the underlying pathology and it is associated with secondary degeneration of axons (Pierpaoli et al., 2001). However, the use of DA and RD remain controversial because a change in RD can cause a fictitious change in DA and vice-versa in voxels characterized by crossing fibers (Wheeler-Kingshott and Cercignani, 2009; Jones et al., 2013).

Different approaches have been used to assess the structural connectivity in DLB patients. They ranged from conventional analyses, which use region of interest (ROI) (Bozzali et al., 2005) or tract-specific method (Ota et al., 2009) to voxel-based approaches, which use statistical parametrical mapping analysis (Lee et al., 2010) or tract-based spatial statistics (Hattori et al., 2012; Watson et al., 2012). However, all these approaches present limitations. The conventional analysis methods are hindered by manual interaction and in particular, the partial volume contamination from adjacent tracts may induce site selection bias, resulting in additional inter-observer variability in the measurements. The voxel-based approach is limited because: (1) no physical characteristics are measured directly; (2) it cannot ensure voxel correspondence of the same tract across subjects (Yeatman et al., 2012); (3) coregistration algorithms do not accurately align fiber tracts which are affected by variation in size and shape (Wassermann et al., 2011). Therefore, the voxel-based approach may not have sufficient precision at the individual level for dementia patient populations, given that patients with dementia are largely affected by brain deformation and substantial variability of long-range fiber tracts morphology among subjects (Wassermann et al., 2011; Yeatman et al., 2012).

TRActs Constrained by UnderLying Anatomy (TRACULA) is a recent tool for automatic reconstruction of a set of major white-matter pathways from diffusion-weighted MR images. It uses global probabilistic tractography with anatomical priors. Prior distributions on the neighboring anatomical structures of each pathway are derived from an atlas and combined with the FreeSurfer cortical parcelation and subcortical segmentation of the subject that is being analyzed to constrain the tractography solutions (Yendiki et al., 2011, 2013). TRACULA has benefits in terms of: (1) overcoming the limitations related to manual interaction, thus facilitating the application of tractography to large studies; (2) measuring, for each patient, the DTI-derived metrics from each tract of interest; (3) overcoming coregistration issues linked to voxel-based approaches (Wassermann et al., 2011; Yendiki et al., 2011, 2013).

In the current study, we used TRACULA to assess structural connectivity in a cohort of DLB and AD patients as well as healthy controls. Our hypothesis was that WM tracts linked to visual, attentional, and mnemonic functions are differentially and selectively affected in DLB and AD.

Because the axonal degeneration can be initiated either by the degeneration of the cell bodies associated with these axons, or by the direct WM injury, we also performed a correlation analysis between WM and gray matter (GM) metrics to assess whether possible structural connectivity alteration in AD and DLB could be the consequence of GM neuronal loss.

## Materials and Methods

### Study Sample

The current research was approved by the local Ethics Committee and was performed according to the Declaration of Helsinki (1997) and subsequent revisions. Data will be made freely available upon request. All subjects (or their caregivers, where appropriate) provided written informed consent. Fourteen DLB and 14 AD patients were recruited from our Memory Clinic and Movement Disorder Clinic. Fifteen age-matched volunteers were recruited from our non-demented case register. AD patients fulfilled the National Institute of Neurological and Communicative Diseases and Stroke/AD and Related Disorders Association criteria (McKhann et al., 1984). Probable DLB diagnosis was based on consensus guidelines (McKeith et al., 2005). As part of their clinical work up, all patients underwent Computerized Tomography or MRI and dopaminergic presynaptic ligand ioflupane SPECT (DAT scan) within 6 months before the inclusion in the study. In addition, all patients were assessed with electroencephalography (EEG) recordings as abnormalities characterized by parieto-occipital dominant frequency alterations have previously been shown to reliably differentiate probable DLB from AD (Bonanni et al., 2008).

### Clinical Assessment

All participants underwent clinical and neuropsychological evaluations. Specifically, Mini Mental State Examination (MMSE) (Folstein et al., 1975), Clinical Dementia Rating (CDR) (Morris, 1993), and Dementia Rating Scale-2 (DRS-2) (Jurica et al., 2001) were used to investigate cognitive deterioration. Frontal Assessment Battery (FAB) (Dubois et al., 2000) and Clinician Assessment of Fluctuations (CAF) (Walker et al., 2000) were included to assess, respectively, the severity of frontal dysfunction and the presence and severity of cognitive fluctuations. Unified Parkinson’s Disease Rating Scale (UPDRS)-motor section III (Fahn and Elton, 1987) assessed the presence and severity of extrapyramidal signs. Neuropsychiatric Inventory (NPI) was used to determine the frequency and severity of any neuropsychiatric features (Cummings et al., 1994). In particular, the NPI item-2 hallucinations assessed the occurrence as well as severity × frequency of visual hallucinations. Presence/absence of REM sleep Behavior Disorder (RBD) was determined according to minimal International Classification of Sleep Disorders (ICSD) criteria (World Health Organization, 1992) and confirmed by polysomnography. Patients were treated with l-DOPA (all DLB patients), rivastigmine or donepezil (all AD and DLB patients with same daily dosages), quetiapine (5 DLB and 5 AD), clozapine (4 DLB), risperidone (4 AD), and clonazepam (14 DLB patients, who presented with RBD).

### MR Data Acquisition

All measurements were carried out with a Philips Achieva 3 T scanner (Philips Medical System, Best, The Netherlands) equipped with eight-channel receiver coil. After scout and reference sequences, three dimensional T_1_-Weighted Turbo Field-Echo (3D T_1_-W TFE, TR/TE = 11/5 ms, slice thickness = 0.8 mm, FOV = 256 mm × 192 mm × 170 mm) and Diffusion-Weighted Image Spin-Echo (DWI-SE; TR/TE = 3691/67 ms, slice thickness of 4 mm, FOV = 230 mm × 230 mm × 139 mm, 15 diffusion-sensitive gradient directions) sequences were performed on all participants. T_2_-weighted fluid attenuation inversion recovery (FLAIR, TR/TE = 11000/125 ms, slice thickness of 4 mm, FOV = 240 mm × 129 mm × 222 mm) sequence was also performed to assess vascular pathology or WM abnormalities.

### Leukoencephalopathy Burden Evaluation

The FLAIR image of each participant was evaluated in blind by two experienced neuroradiologists in two independent sessions. Intra- and inter-rater reliability tests were performed by non-parametric Kruskal–Wallis test, followed, respectively, by Wilcoxon and Mann–Whitney *post hoc* test to allow comparisons within and between groups.

The rating scale described in Fazekas et al. (1987) was used to assess the different types of hyperintense signal abnormalities in the deep white matter (DHWM). Specifically, DHWM was scored as 0 = absent, 1 = punctate foci, 2 = beginning confluence of foci, 3 = large confluent areas.

### Gray Matter Morphometry

Structural T_1_-weighted and DWI images were processed by using Freesurfer processing stream (Fischl and Dale, 2000; Yendiki et al., 2011).^1^ By using recon-all command line, we performed the automated reconstruction and labeling of cortical and subcortical regions [classified by using the Desikan–Killiany Atlas (Desikan et al., 2006)] on the high-resolution anatomical T_1_-weighted images of each subject. Subcortical volumes and mean thickness of each cortical region were extracted by using “asegstats2table” and “aparcstats2table” command line.

### Structural Connectivity Analysis

The DWI image of each subject was corrected from distortions induced by eddy currents and motion (Yendiki et al., 2013). Next, intra-subject registration between the individual’s low-b diffusion and T_1_ images was performed by using an affine registration method that seeks to maximize the intensity contrast of the *b* = 0 image across the cortical gray/white boundary, which is obtained from the T_1_-images. Subsequently, affine registration was carried out between each individual’s structural MRI image and MNI152-1mm atlas. WM mask was created by extracting the cerebral WM, cerebellar WM, ventral diencephalon, and brainstem from the individual’s FreeSurfer cortical parcelation and subcortical segmentation (obtained by recon-all command line). Cortical mask was obtained by mapping the cortical parcelation labels to the volume, growing them into the WM by 2 mm and combining all the grown cortical labels into a mask. Anatomical brain mask was produced by binarizing and dilating the entire cortical parcelation and subcortical segmentation. All the above masks were obtained from individual T_1_ space to individual diffusion space and to the template space. Least-squares tensor estimation was carried out using FSL’s (FMRIB’s Diffusion Toolbox^2^) and mapping all scalar output volumes of the tensor fit (FA, MD, DR, and DA) from diffusion space to the template space. Combining the atlas data with the previously obtained individual’s masks, pathways were computed in template space. The TRACULA atlas data were used to estimate *a priori* probabilities that each pathway intersects each of the labels in the cortical parcelation and subcortical segmentation, at each point along the pathway’s trajectory. The atlas set was also used to obtain ROIs for the two endings of each pathway, as well as an initial guess of the location of the control points of each pathway, to be used in the subsequent pathway reconstruction. After estimation of pathway priors, ball-and-stick model fitting was performed. Estimation of the *a posteriori* probability distribution of the location of each pathway in the individual and reconstructed volumetric distributions was performed for corticospinal tract, inferior longitudinal fasciculus, uncinate fasciculus, ATR, cingulum-cingulate gyrus (CCG) (supracallosal) bundle, cingulum-angular (infracallosal) bundle (CAB), superior longitudinal fasciculus-parietal bundle, superior longitudinal fasciculus-temporal bundle, corpus callosum-forceps major, and corpus callosum-forceps minor. With the exception of corpus callosum-forceps major and corpus callosum-forceps minor, which are inter-hemispheric connections, all other pathways were labeled for the left and right hemisphere. We therefore defined a total of 18 tracts per subject. From each one, DTI metrics (FA, MD, RD, and DA) were averaged over an entire pathway.

### Target Regions Definition

For each WM tract showing significant difference among groups, we defined the its target regions. Specifically, for the right ATRs, the target regions were the right mediodorsal nuclei of thalami and the areas within the right frontal lobe (caudal-anterior cingulate gyrus, caudal-middle frontal gyrus, lateral-orbitofrontal gyrus, medial-orbitofrontal gyrus, parsopercularis, parsorbitalis, parstriangularis, rostralanteriorcingulate gyrus, rostral-middle frontal gyrus, superior frontal gyrus, frontal pole); for the left and right CABs, the target regions were the ipsilateral regions within the temporal lobe (entorhinal and parahippocampal cortices, hippocampus, inferiortemporal gyrus, middle temporal gyrus, superior temporal gyrus (STG), temporal pole, transverse temporal gyrus, insula) and ipsilateral precuneus and posterior cingulate cortex (PCC); for the left CCG bundles, the target regions were the areas within the left frontal lobe and left precuneus and PCC; for the right and left inferior longitudinal fascicles, the target regions were the ipsilateral areas within temporal and occipital (cuneus, fusiform gyrus, lateral occipital gyrus, lingual gyrus and pericalcarine cortex) lobes; for the right and left uncinate fasciculus, the target regions were the ipsilateral regions within frontal and temporal lobes. All cortical areas were defined by using the Desikan–Killiany Atlas.

### Microstructural Assessment of Thalamic Regions

Microstructural assessment of the thalamic regions were performed by using Functional MRI of the Brain (FMRIB) Software Library (FSL) version 4.1 (Smith et al., 2004,^3^). In detail, for each subject, noise reduction was carried out using Smallest Univalue Segment Assimilating Nucleus (SUSAN) algorithm on structural images and eddy-currents correction on diffusion images. Brain Extraction Tool (BET) was carried out for brain and skull extraction of the structural and DWI images. T_1_-W structural image of each subject was co-registered in common space on the non-linear MNI152 template with 1 mm × 1 mm × 1 mm resolution, by means of affine transformations based on 12 degree of freedom (three translations, three rotations, three scalings, and three skews) using FMRIB’s Linear Image Registration Tool (FLIRT). FMRIB’s Integrated Registration and Segmentation Tool (FIRST) was used to automatically segment thalami (Patenaude et al., 2011). Thalami masks were obtained by binarizing the FIRST outputs. The DTI maps were registered to MNI standard space using: (1) FLIRT to register each subject’s b0 image to its native structural image, and (2) FMRIB’s non-linear registration tools to register the structural and diffusion images to MNI space (1 mm × 1 mm × 1 mm). Next, “fslroi command line” was used to overlap the thalami masks on MD maps and to minimize the misalignment between DWI and structural images. Oxford thalamic connectivity atlas (provided by FSL tool) was adapted on the thalami masks to define the medio-dorsal nuclei projecting to frontal cortex (Figure S1 in Supplementary Material). To exclude thalamic voxels that contained cerebrospinal fluid (CSF), the MD images were segmented using FMRIB’s Automated Segmentation Tool (FAST) and CSF binarized to be used as exclusion mask. To exclude voxels out of the thalamic range, manual editing was applied where needed. Finally, MD values were calculated within the connectivity-defined subregion (Delli Pizzi et al., 2015).

### Statistical Analysis

One-way ANOVA and Bonferroni *post hoc* test was also performed on demographic and clinical data. Chi-squared test was carried out for sex. Kruskal–Wallis one-way analysis of variance by ranks was used to assess group difference on DHWM. *T*-Tests (independent samples) were applied on TRACULA outcomes to test the differences among groups (AD vs. controls, DLB vs. Controls and AD vs. DLB). Bonferroni’s correction was applied to adjust the *p*-level (corrected *p* threshold was set at 0.05/18 tracts = 0.003). Analysis of covariance (ANCOVA) was performed to exclude the possible effect of DHWM on DTI findings.

Within each patient group, linear regression was performed to assess the relationship between: (1) DTI metrics within tract of interest (dependent variable) and our primary clinical measures (independent variables: age, DHWM, FAB, MMSE, NPI hallucination-item, UPDRS scores); (2) DTI metrics (dependent variable) and the GM measures within target regions of each tract of interest (independent variables); age, DHWM and mean cortical thickness value of each hemisphere were added to regressor as nuisance factors. If any significant relationship exits between WM and GM metrics, we assume that the WM changes are consequence of GM neuronal loss (Huang et al., 2012).

To ensure the specific effect of GM patterns of atrophy on DTI metrics, a further regression analysis was also performed including DTI metrics as dependent variable and the thickness of non-target regions as independent variables.

## Results

### Demographic and Clinical Features

Demographic features and neuropsychological test scores were summarized in Table 1.

**Table 1:** **Demographic and clinical features**.

Characteristics	DLB	AD	Controls
Number of subjects/patients	14	14	15
Age^a^,^b^	75.8 ± 3.8	75.4 ± 6.2	75.0 ± 4.8
Male gender (in percentage)^a^,^c^	50.0	50.0	46.7
Disease duration (years)^d^	3.1 ± 0.6	3.0 ± 0.7	–
Education level (years)^a^,^e^	7 ± 4	7 ± 4	7 ± 3
CDR^a^,^f^	2.04 ± 0.50	1.93 ± 0.47	–
MMSE^a^,^g^	18.0 ± 4.9	18.1 ± 4.6	27.7 ± 0.6
DRS^a^,^h^	93.3 ± 17.8	84.6 ± 13.5	136.8 ± 0.86
FAB^a^,^i^	6.1 ± 3.0	6.7 ± 3.1	17.1 ± 1.0
DHWM^j^	1.36 ± 0.74	1.14 ± 0.66	0.80 ± 0.41
CAF	4.5 ± 2.5	0.0 ± 0.0	0.0 ± 0.0
UPDRS III	26.1 ± 9.2	0.0 ± 0.0	0.0 ± 0.0
NPI item-2 hallucinations	4.1 ± 1.5	0.0 ± 0.0	0.0 ± 0.0

*Values are expressed as mean ± SD*.

*^a^The *p*-values were calculated using the one-way ANOVA; Bonferroni *post hoc* test was also performed when *F*-test was significant*.

*^b^Main interaction among groups: *F*_2,42_ = 0.743, *p* = 0.482*.

*^c^The *p*-values were calculated using chi-squared test: $\chi_{1}^{2}=0.47,p=0.977$*.

*^d^The *p*-values were calculated using the independent-samples *t*-test: *t*_26_ = −0.291, *p* = 0.773*.

*^e^Main interaction among groups: *F*_2,42_ = 0.92, *p* = 0.912*.

*^f^The *p*-values were calculated using the independent-samples *t*-test: *t*_26_ = 0.045; *p* = 0.565*.

*^g^Main interaction among groups: *F*_2,42_ = 30.435, *p* < 0.001; *post hoc*: controls vs. AD, *p* < 0.001; controls vs. DLB, *p* < 0.001 and AD vs. DLB, *p* = 1.000*.

*^h^Main interaction among groups: *F*_2,41_ = 70.276, *p* < 0.001; *post hoc*: controls vs. AD, *p* < 0.001; controls vs. DLB, *p* < 0.001 and AD vs. DLB, *p* = 0.242*.

*^i^Main interaction among groups: *F*_2,42_ = 88.905, *p* < 0.001; *post hoc*: controls vs. AD, *p* < 0.001; controls vs. DLB, *p* < 0.001 and AD vs. DLB, *p* = 1.000*.

*^j^Kruskal–Wallis main interaction among groups: $\chi_{2}^{2}=4.985,P=0.083$*.

*AD, Alzheimer’s Disease; CAF, clinician assessment of fluctuations; DLB, dementia with Lewy bodies; CDR, Clinical Dementia Rating, DRS, Dementia Rating Scale; FAB, frontal assessment battery; MMSE, mini mental state examination; NPI, neuropsychiatric inventory; UPDRSIII, Unified Parkinson’s Disease Rating Scale-motor section III; DHWM, hyperintense signal abnormalities in the deep white matter*.

No differences in terms of age, sex, and educational level were observed among groups.

No differences on global test of cognition (DRS-2, MMSE, CDR) and on the severity of frontal dysfunction (FAB score) were found between AD and DLB patients. All DLB patients had RBD. All DLB patients had visual hallucinations and cognitive fluctuations. None of the AD patients had visual hallucinations or cognitive fluctuations, as expected given inclusion criteria. All DLB patients showed an abnormal quantitative EEG pattern profile consistent with a DLB diagnosis (Bonanni et al., 2008) and represented by slow dominant frequency (in the theta and pre-alpha band) in posterior leads and a dominant frequency variability >1.5 Hz. None of the AD patients or controls showed these DLB-specific EEG characteristics (Bonanni et al., 2008). Dopamine-transporter hypocaptation in the caudate nuclei at SPECT-DAT scan was observed in all DLB patients (bilateral in 12). SPECT-DAT scan abnormalities were not observed in AD patients or control subjects.

### White Matter Hyperintensity Evaluation

Intra- and inter-rater reliability test showed no differences in the evaluation of white matter hyperintensity (*p* = 1.000).

No significant difference on DHWM was found among groups (Table 1).

In DLB, DHWM was absent (score = 0) in one patient, was present with punctate foci (score = 1) in eight patients, with beginning confluence of foci (score = 2) in four patients and with large confluent areas (score = 3) in one patients. In AD, DHWM was absent (score = 0) in one patient, was present with punctate foci (score = 1) in 11 patients, with the beginning confluence of foci (score = 2) in one patients and evidence of large confluent areas (score = 3) in one patient. In controls, DHWM was absent (score = 0) in three subjects and was present with punctate foci (score = 1) in 12 subjects.

### Structural Connectivity

**Figure 1:**
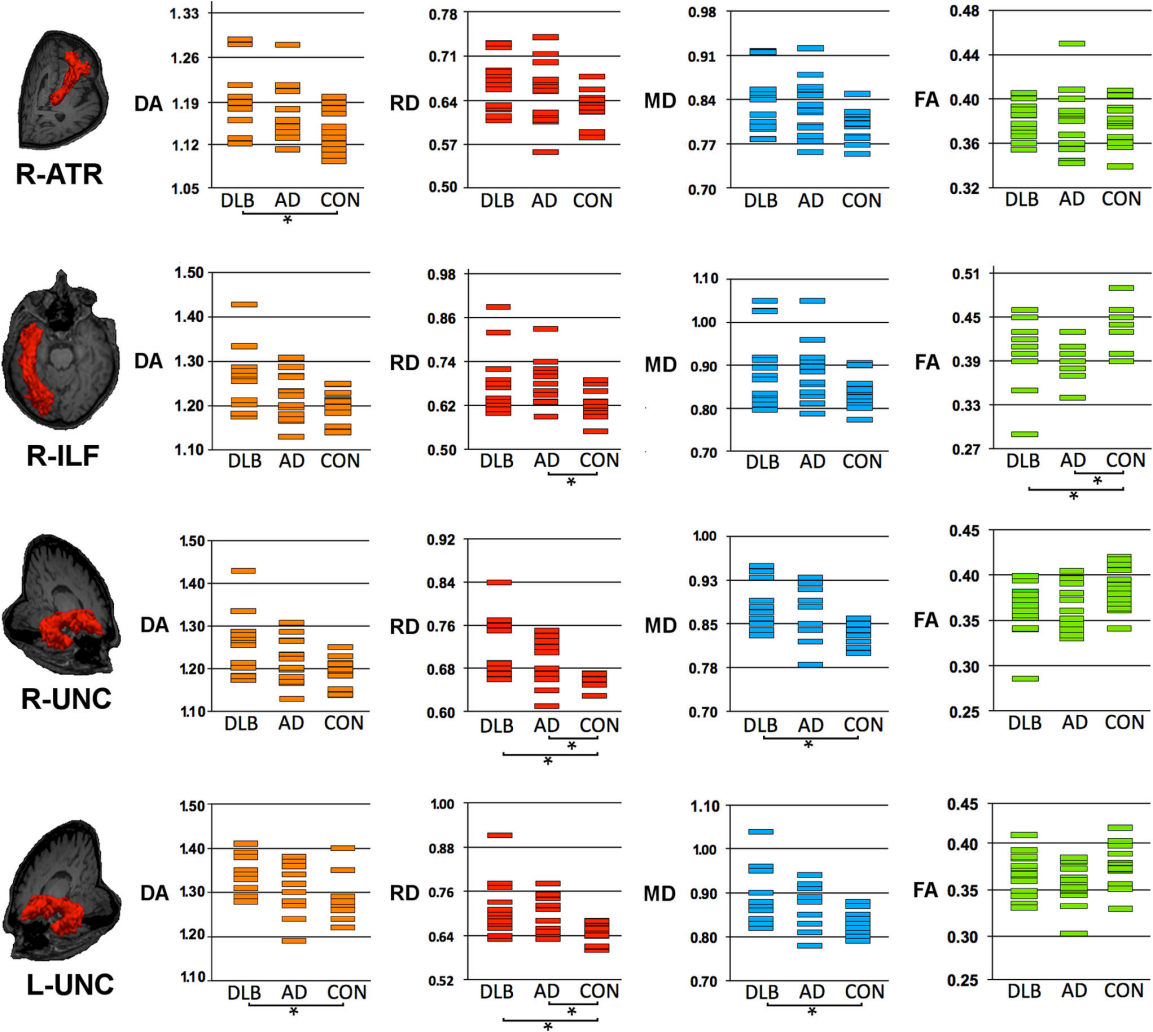
**Structural connectivity showing DTI-metrics values within right anterior thalamic radiation (ATR), right inferior longitudinal fascicle (ILF) and right and left uncinate fasciculus (UNC)**. Representative images showing TRACULA output: the tract of interest is colored in red and overlaid on individual’s structural image. The distribution of DTI-metrics values within each tract of interest and within group is reported in the scatter-plots: orange, red, blue, and green rectangles represent axial diffusivity (DA), radial diffusivity (RD), mean diffusivity (MD) and fractional anisotropy (FA), respectively. Significant differences between groups are marked with dark lines and asterisks. The values of DA, RD, and MD are reported as values ×10^−3^mm^2^/s. AD, Alzheimer’s Disease; DLB, dementia with Lewy bodies; R, right; L, left.

**Figure 2:**
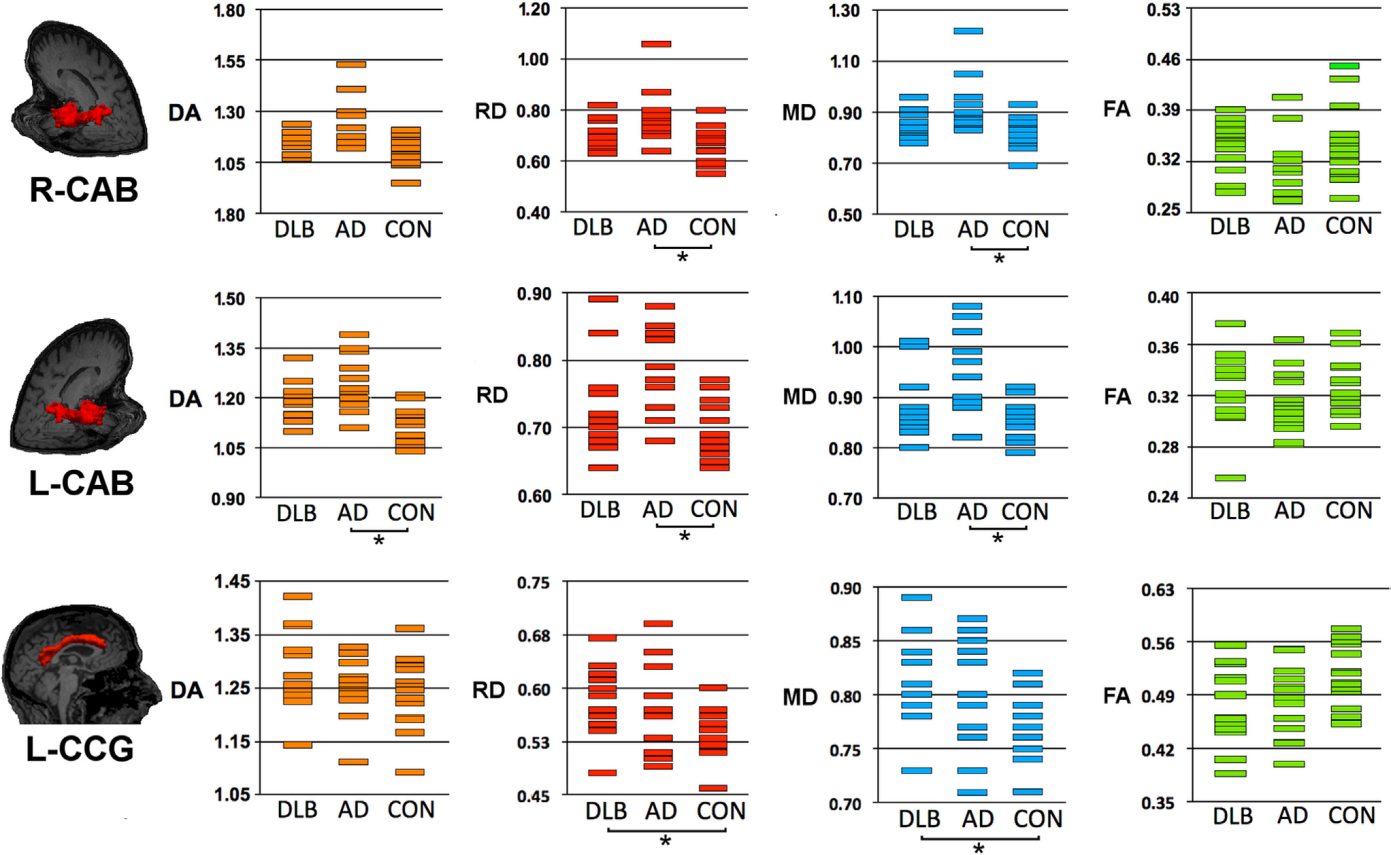
**Structural connectivity showing DTI-metrics values for left cingulum-angular (infracallosal) cingulum-angular bundle (CAB) and bilateral cingulum-cingulate gyrus (supracallosal) bundle (CCG)**. Representative images showing TRACULA output: the tract of interest is colored in red and overlaid on individual’s structural image. The distribution of DTI-metrics values within each tract of interest and within group is reported in the scatter-plots: orange, red, blue, and green rectangles represent axial diffusivity (DA), radial diffusivity (RD), and mean diffusivity (MD), respectively. Significant differences between groups are marked with dark lines and asterisks. The values of DA, RD, and MD are reported as values ×10^−3^mm^2^/s. AD, Alzheimer’s disease; DLB, dementia with Lewy bodies; R, right; L, left.

Table 2 and Figures 1 and 2 summarize significant results obtained from DTI analysis by TRACULA. Tables S1–S4 in Supplementary Material provide the complete (significant and non-significant) statistical results for each metric and tract. Table S5 in Supplementary Material shows the results of ANCOVA analyses, which excluded the possible effect of DHWM on DTI results.

**Table 2:** **Mean DTI-metrics values of left and right white matter tracts for each group**.

Metric	Tract	DLB	AD	Controls	DLB vs. controls	AD vs. controls	DLB vs. AD
FA	R-ILF	0.40 ± 0.04	0.40 ± 0.03	0.44 ± 0.02	***p* = 0.002**	***p* = 0.000**	*p* = 1.000
MD^a^	L-CAB	0.88 ± 0.06	0.94 ± 0.08	0.84 ± 0.04	*p* = 0.055	***p* = 0.000**	*p* = 0.020
	R-CAB	0.86 ± 0.05	0.92 ± 0.10	0.81 ± 0.06	*p* = 0.038	***p* = 0.001**	*p* = 0.041
	L-CCG	0.82 ± 0.04	0.80 ± 0.05	0.77 ± 0.03	***p* = 0.001**	*P* = 0.042	*p* = 0.383
	L-UNC	0.89 ± 0.06	0.86 ± 0.05	0.82 ± 0.03	***p* = 0.001**	*p* = 0.010	*p* = 0.198
	R-UNC	0.89 ± 0.06	0.87 ± 0.04	0.84 ± 0.02	***p* = 0.001**	*p* = 0.011	*p* = 0.244
RD^a^	L-CAB	0.73 ± 0.07	0.79 ± 0.08	0.70 ± 0.04	*p* = 0.216	***p* = 0.000**	*p* = 0.022
	R-CAB	0.71 ± 0.06	0.77 ± 0.10	0.67 ± 0.07	*p* = 0.097	***p* = 0.002**	*p* = 0.038
	L-CCG	0.59 ± 0.05	0.58 ± 0.06	0.53 ± 0.03	***p* = 0.001**	*p* = 0.017	*p* = 0.583
	R-ILF	0.68 ± 0.08	0.68 ± 0.06	0.63 ± 0.03	*p* = 0.021	***p* = 0.003**	*p* = 0.982
	L-UNC	0.72 ± 0.07	0.69 ± 0.05	0.64 ± 0.02	***p* = 0.001**	***p* = 0.002**	*p* = 0.310
	R-UNC	0.71 ± 0.06	0.70 ± 0.04	0.66 ± 0.01	***p* = 0.001**	***p* = 0.001**	*p* = 0.471
DA^a^	R-ATR	1.20 ± 0.05	1.18 ± 0.05	1.14 ± 0.04	***p* = 0.001**	*p* = 0.009	*p* = 0.256
	L-CAB	1.19 ± 0.06	1.24 ± 0.08	1.12 ± 0.06	*p* = 0.006	***p* < 0.001**	*p* = 0.048
	R-CAB	1.16 ± 0.04	1.22 ± 0.12	1.11 ± 0.07	*p* = 0.027	*p* = 0.005	*p* = 0.091
	L-UNC	1.24 ± 0.05	1.21 ± 0.05	1.18 ± 0.04	***p* = 0.002**	*p* = 0.191	*p* = 0.075

*^a^Values ×10^−3^ mm^2^/s*.

*Table reports the DTI-metrics values (mean ± SD) and the statistical outcomes (derived from *t*-test for independent samples and Bonferroni’s correction) for tracts showing significant differences among groups. Bold characters indicate statistically significant results. The complete statistical results for each metric and tract were reported in Tables S1–S4 in Supplementary Material*.

*AD, Alzheimer’s Disease; DLB, dementia with Lewy bodies; FA, fractional anisotropy; MD, mean diffusivity; RD, radial diffusivity; DA, axial diffusivity; ATR, anterior thalamic radiation, CAB, cingulum-angular (infracallosal) bundle, CCG, cingulum-cingulate gyrus (supracallosal) bundle, ILF, inferior longitudinal fasciculus, UNC, uncinate fasciculus*.

As compared with controls, DTI-metric changes in DLB were found in the right ATR (DA), right inferior longitudinal fascicule (FA), left CCG bundle (RD), right (RD, MD), and left (DA, RD, MD) uncinate fasciculus.

As compared with controls, DTI-metric changes in AD were found in the right (RD, MD) and left (DA, RD, MD) CAB, right inferior longitudinal fascicule (FA, RD), and right (RD) and left (RD) uncinate fasciculus.

No significant difference was found between DLB and AD.

No correlation was found between WM metrics and clinical outcomes.

### Relationship Between White Matter and Gray Matter Metrics

**Figure 3:**
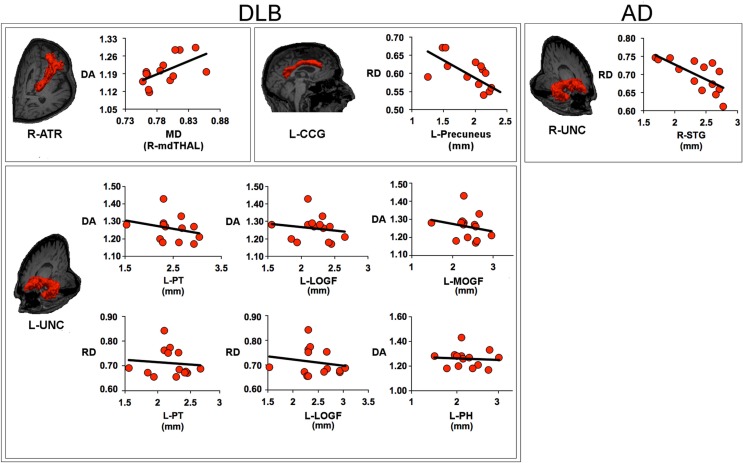
**Scatter plots describe the relationship between the metrics within the white matter tract and the measures of gray matter within its target regions**. The values of axial diffusivity (DA), radial diffusivity (RD), and mean diffusivity (MD) are reported as values ×10^−3^mm^2^/s. For DLB patients, the panels show: the relationship between DA within the right anterior thalamic radiation (ATR) and MD within the right medio-dorsal thalamic region (mdTHAL), which projects to prefrontal cortex; the relationship between RD within the left cingulum-cingulate gyrus (supracallosal) bundle (CCG) and the thickness of left precuneus; the relationship between DA within the left uncinate fasciculus (UNC) and the thickness of left pars triangularis (PT), left medial orbitalfrontal gyrus (MOFG), left lateral orbitofrontal gyrus (LOFG) and between the RD within the left UNC and the thickness of left PT and LOFG; for AD patients, the panel shows the relationship between RD within the right uncinate fasciculus and the thickness of right superior temporal gyrus (STG). AD, Alzheimer’s disease; DLB, dementia with Lewy bodies; R, right; L, left.

Figure 3 shows the relationship between white matter and GM metrics.

Within the DLB group, the increase of DA values in the right ATR was correlated to MD values in the mediodorsal nuclei of the right thalamus (*t* = 2.487, β = 0.583, *p* = 0.029); the increase of RD values in the left CCG bundle was anti-correlated to cortical thickness in the left precuneus (*t* = −3.028, β = −0.658, *p* = 0.010); the increase of DA values in the left uncinate fasciculus was anti-correlated to cortical thickness in the left mediorbitofrontal gyrus (*t* = −7.902, β = −1.087, *p* < 0.001), left laterorbitofrontal gyrus (*t* = 8.840, β = 1.171, *p* < 0.001), left pars triangularis (PT) (*t* = −6.712, β = −0.977, *p* < 0.001), left parahippocampus (*t* = 3.958, β = 0.466, *p* = 0.003); the increase of RD values in the left uncinate fasciculus was anti-correlated to cortical thickness in the left laterorbitofrontal gyrus (*t* = −4.719, β = −1.266 *p* = 0.001) and left PT (*t* = 2.650, β = 0.711, *p* = 0.023).

Within the AD group, no significant correlation was found between DTI metrics in the right and left CCG bundle and GM measure in the target regions; the increase of DA values within the right uncinate fasciculus was correlated to right STG thickness (*t* = −3.427, β = −0.703, *p* = 0.005).

No correlations were found between FA values in the right inferior longitudinal fascicule and cortical thickness within temporal and occipital lobes.

No significant relationship was found between the DTI metrics and the cortical thickness in the non-target regions.

## Discussion

In this study, we found specific pattern of WM alterations in DLB and AD.

As compared with controls, the ATR was altered in DLB but not in AD.

This tract connects the dorso-medial thalamic nuclei with the prefrontal cortex (Wakana et al., 2007; Yendiki et al., 2011). The fronto-thalamic connectivity plays a relevant role in consciousness (Ward, 2011) and alertness (Tomasi et al., 2009). Recently, structural and functional alteration of fronto-thalamic loop has been described in DLB patients (Kenny et al., 2013; Delli Pizzi et al., 2014a, 2015). In the current study, we found a close relationship between microstructural GM damage within thalamic nuclei projecting to frontal lobe and secondary axonal degeneration (expressed by DA) within ATR. In addition, we did not find any correlation between frontal thickness and fronto-thalamic tract alterations. Thus, we suggest that the secondary axonal degeneration within the ATR could be linked by GM neuronal loss in the thalami. Of note, the synchronized activity of the thalamo-cortical pathway modulates the information flow necessary for conscious cognitive processes (León-Domínguez et al., 2013). However, we did not find a significant correlation between the ATR degeneration in DLB patients and the CAF scores. Although the CAF questionnaire remains validated as a measure of the frequency and duration of the cognitive fluctuations and has been used in numerous studies as a metric to examine the pathophysiological basis of cognitive fluctuations (McKeith et al., 2005; Bonanni et al., 2008; Taylor et al., 2012), it has been superseded by recent scales which may have better diagnostic utility in distinguishing flCog in DLB compared with AD (e.g., dementia cognitive fluctuation scale, Lee et al., 2014). Therefore, further studies by using more recent neuropsychological tests are warranted to investigate whether the fronto-thalamic structural connectivity alteration in DLB could be relevant to explain the impairment of the cognitive processes (necessary for consciousness) in DLB.

In line with literature (Bozzali et al., 2005; Kantarci et al., 2010; Lee et al., 2010), the inferior longitudinal fascicle was affected in both forms of dementia as compared with controls. This tract is a ventral associative bundle transmitting visual information from occipital areas to the temporal lobe (Catani et al., 2012). It plays an important role in visual object recognition and it is strongly implicated in disorder of visual perception (Catani et al., 2012). The degeneration of inferior longitudinal fascicle has been related to visual hallucinations in DLB patients (Kantarci et al., 2010). However, in the current study, we did not observe a significant relationship between WM damage within inferior longitudinal fascicle and the frequency and severity of visual hallucinations. Recent models and different neuroimaging studies on DLB have suggested that visual hallucinations could be more reliant upon dorsal network impairment (Taylor et al., 2012; Delli Pizzi et al., 2014b; Shine et al., 2014) and further investigation of this in DLB patients is warranted and whether these possible alterations were related to visual hallucinations. However, a limitation of our study, TRACULA does not allow the assessment of the dorsal visual pathway in its entirety. In particular, it is not able to detect the first and second branches of superior longitudinal fascicle (Yendiki et al., 2011), which are involved in visuo-spatial attention (Thiebaut de Schotten et al., 2011).

As compared to controls, the CCG (supracallosal bundle) was damaged in DLB but not in AD. The CCG bundles wrap around the corpus callosum from medial frontal cortex and anterior cingulate cortex to dorsal PCC (Greicius et al., 2009). Input from the frontal lobes modulates the level of dorsal posterior cingulated cortex activity, and consequently the top-down and bottom-up attentional signals (Bonnelle et al., 2012). In this way, the CCG bundles regulates the attentional focus, influencing the “metastability” of the brain as a whole and shifting the balance of attention along an internal/external and broad/narrow dimension (Leech and Sharp, 2014). Furthermore, the loss of the normal top-down cortico-cortical communication from the dorsal anterior cingulate cortex to the dorsal PCC has been associated to alterations in arousal and awareness (Horovitz et al., 2008, 2009; Larson-Prior et al., 2009; Boly et al., 2012). In this study, we found a relationship between the secondary processes of neurodegenration with supracallosal bundle (expressed by RD) and the thickness of the dorsal precuneus. Hence, the axonal neurodegeration of supracallosal bundle, probably related to demyelination process, could be linked to neuronal loss in the posterior cortical regions, which are relevant in DLB (Delli Pizzi et al., 2014b) and attention processing (Leech and Sharp, 2014).

As compared to controls, the angular (infracallosal) bundle was damaged in AD but not in DLB. The CAB connects the ventral PCC to temporal structures including the perforant path (the main input to the hippocampus, extending from the entorhinal cortex to dentate gyrus) and several other fibers reaching entorhinal cortex, parahippocampal gyrus, and associated areas (Thiebaut de Schotten et al., 2011; Leech and Sharp, 2014). The temporal structures (Jack et al., 1999; Janke et al., 2001) and the ventral PCC are highly affected in AD (Buckner et al., 2008). Particularly, metabolic abnormalities within these regions are related to amyloid deposition and to brain atrophy in a spatial distribution that reflects the default-mode network (Greicius et al., 2009). Furthermore, it was also observed that the functional connectivity within the default-mode network is reduced between PCC and hippocampal areas and this tract is particularly involved in internally directed cognition such as memory retrieval and planning, which are prevalently and prominently affected in AD (Buckner et al., 2008; Greicius, 2008).

Although AD affects primarily GM, WM disruption is also widespread (Scheltens et al., 1995; Smith et al., 2000; Gouw et al., 2008; Huang et al., 2012). In the current study, the profiles of DTI-metric changes within infracallosal bundle of AD patients and the poor correlation between WM and GM alteration suggest that heterogeneous pathologic processes such as axonal damage and breakdown of oligodendrocytes and myelin could be independent from neuronal loss in the cortex and subcortical structures.

Uncinate fasciculus was affected in both AD and DLB as compared to controls. This tracts connects the anterior temporal lobe with the orbital and polar frontal lobe including orbitofrontal area and inferior frontal gyrus (Catani et al., 2012). Uncinate fasciculus functions are linked to episodic memory, language and social-emotional processing (Catani et al., 2012). Its disruption has been reported in both AD and DLB (Serra et al., 2012). However, its contribution to these forms of dementia is still unclear. In the current study, we found a relationship between DTI metrics within uncinate fasciculus and the cortical thickness of (1) the PT and of the medio- and lateral-orbitofrontal gyrus in DLB patients and (2) the STG in AD patients. These findings suggest that the structural alteration within uncinate fasciculus of AD and DLB could be caused by cortical neuronal loss more than by direct WM injury. This hypothesis is in agreement with a recent paper by Serra et al. (2012), suggesting that the uncinate fascicle damage could be linked to GM atrophy in the medial temporal lobe structures and to memory impairment in AD and with prominent involvement of the frontal lobes in DLB.

In conclusion, different patterns of WM alteration were found in AD and DLB. In particular, the structural connectivity is affected within fronto-thalamic and fronto-parietal attentional network in DLB and within mnemonic pathways in AD. Furthermore, the high correlation between GM and WM metrics within ATR, supracallosal bundle and uncinate fasciculus suggests that the structural connectivity alteration in DLB could be linked to GM neuronal loss rather than by direct WM injury. Thus, this finding supports the key role of cortical and subcortical atrophy in DLB.

We must acknowledge that due to low sample size the proposed structural MRI protocol cannot be applied for clinical purposes, i.e., for differential diagnosis between DLB and AD, which would require the replication of the study on larger cohorts by different centers.

## Author Contributions

Study design: LB, SP, MO. Subjects and data collection: LB, SP, MO, RF. Data Analysis: SP, RE, AT. Data interpretation: LB, SP, J-PT, MO, RF. Paper Drafting: SP, LB. Paper revising: RF, J-PT, RE, AT, AT, MO. Final approval of the version to be published: SP, RF, J-PT, RE, AT, AT, MO, LB. Agreement to be accountable for all aspects of the work in ensuring that questions related to the accuracy or integrity of any part of the work are appropriately investigated and resolved: SP, RF, J-PT, RE, AT, AT, MO, LB.

## Conflict of Interest Statement

The authors declare that the research was conducted in the absence of any commercial or financial relationships that could be construed as a potential conflict of interest.

## Footnotes

^1^http://ftp.nmr.mgh.harvard.edu

^2^http://fsl.fmrib.ox.ac.uk/fsl/fslwiki/FDT

^3^http://www.fmrib.ox.ac.uk/fsl
