# Supplementary material for: Structural Connectivity is Differently Altered in Dementia with Lewy Body and Alzheimer’s Disease
Source: Front Aging Neurosci. 2015 Nov 2;7:208. doi: 10.3389/fnagi.2015.00208 (PMC4629464; doi:10.3389/fnagi.2015.00208)
Supplement: Supplementary file 1 [file Data_Sheet_1.PDF]

SUPPLEMENTARY MATERIALS

Supplementary table 1. Fractional anisotropy (FA) values for left and right white matter tracts.

Tract	DLB	AD	Controls	DLB vs.		AD vs.		DLB vs. AD	
				t_{27}	p	t_{27}	p	t_{26}	p
FMAJ	0.50±0.07	0.51±0.07	0.56±0.05	-2.704	0.012	-2.250	0.033	-0.333	0.742
FMIN	0.43±0.03	0.43±0.05	0.43±0.05	-0.164	0.871	-0.439	0.664	0.348	0.731
L-ATR	0.38±0.01	0.39±0.03	0.39±0.02	-0.357	0.724	0.622	0.539	-1.003	0.325
R-ATR	0.38±0.02	0.38±0.03	0.38±0.02	0.184	0.855	0.290	0.774	-0.160	0.874
L-CAB	0.33±0.03	0.31±0.02	0.33±0.02	-0.005	0.996	-1.495	0.147	1.206	0.239
R-CAB	0.34±0.04	0.31±0.04	0.34±0.05	-0.295	0.771	-1.783	0.086	1.769	0.089
L-CCG	0.48±0.05	0.48±0.04	0.52±0.04	-2.474	0.020	-2.399	0.024	-0.162	0.873
R-CCG	0.44±0.05	0.45±0.03	0.48±0.05	-1.678	0.105	-1.653	0.110	-0.381	0.706
L-CST	0.49±0.04	0.51±0.02	0.51±0.02	-1.998	0.056	0.103	0.919	-2.124	0.043
R-CST	0.48±0.04	0.49±0.03	0.49±0.02	-1.097	0.282	-1.025	0.314	-0.239	0.813
L-ILF	0.42±0.04	0.43±0.04	0.44±0.03	-1.630	0.115	-1.205	0.239	-0.360	0.722
R-ILF	0.40±0.04	0.40±0.03	0.44±0.02	**-3.419**	**0.002**	**-4.872**	**0.000**	0.000	1.000
L-SLFP	0.38±0.03	0.40±0.02	0.40±0.03	-1.639	0.113	-0.549	0.588	-1.244	0.224
R-SLFP	0.38±0.03	0.38±0.02	0.39±0.02	-1.062	0.298	-0.800	0.431	-0.388	0.701
L-SLFT	0.41±0.04	0.43±0.03	0.43±0.03	-1.631	0.115	0.628	0.535	-2.243	0.034
R-SLFT	0.39±0.03	0.40±0.02	0.40±0.03	-1.526	0.139	-0.193	0.849	-1.476	0.152
L-UNC	0.36±0.03	0.36±0.03	0.39±0.02	-2.553	0.017	-2.480	0.020	-0.145	0.886
R-UNC	0.37±0.02	0.36±0.02	0.37±0.02	-0.969	0.341	-2.003	0.055	0.983	0.335

Bold characters indicate statistically significant results after t-test for indipendent samples and Bonferroni's correction (p threshold 0.05/18=0.003).

Abbreviations: AD=Alzheimer's Disease; DLB=dementia with Lewy bodies; ATR=anterior thalamic radiation, CAB=cingulum-angular (infracallosal) bundle, CCG=cingulum-cingulate gyrus (supracallosal) bundle, CTS=corticospinal tract, ILF=inferior longitudinal fasciculus, SLFP=superior longitudinal fasciculus-parietal bundle, SLFT=superior longitudinal fasciculus-temporal bundle, UNC=uncinate fasciculus, FMAJ=corpus callosum-forceps major, FMIN=corpus callosum-forceps minor.

Supplementary table 2. Mean diffusivity (MD) values for left and right white matter tracts.

Tract	DLB	AD	Controls	DLB vs.		AD vs.		DLB vs. AD	
				t_{27}	p	t_{27}	p	t_{26}	p
FMAJ	0.99±0.16	0.97±0.14	0.89±0.06	2.305	0.029	2.152	0.041	0.325	0.748
FMIN	0.95±0.10	0.93±0.10	0.92±0.08	0.845	0.406	0.375	0.710	0.428	0.672
L-ATR	0.84±0.05	0.83±0.06	0.80±0.02	2.602	0.015	1.731	0.095	0.468	0.644
R-ATR	0.84±0.05	0.83±0.04	0.80±0.02	3.067	0.004	1.880	0.075	0.987	0.333
L-CAB	0.88±0.06	0.94±0.08	0.84±0.04	2.007	0.055	**4.516**	**0.000**	-2.489	0.020
R-CAB	0.86±0.05	0.92±0.10	0.81±0.06	2.183	0.038	**3.555**	**0.001**	-2.148	0.041
L-CCG	0.82±0.04	0.80±0.05	0.77±0.03	**3.545**	**0.001**	2.137	0.042	0.886	0.383
R-CCG	0.81±0.06	0.81±0.06	0.76±0.04	2.939	0.007	2.751	0.010	0.100	0.921
L-CST	0.80±0.08	0.77±0.04	0.74±0.03	2.380	0.025	1.722	0.097	1.176	0.250
R-CST	0.79±0.06	0.79±0.07	0.75±0.03	2.330	0.028	1.649	0.111	0.273	0.787
L-ILF	0.90±0.07	0.89±0.06	0.85±0.04	2.665	0.013	2.679	0.012	0.162	0.872
R-ILF	0.88±0.08	0.88±0.06	0.84±0.03	1.946	0.062	2.188	0.038	0.021	0.984
L-SLFP	0.81±0.08	0.80±0.05	0.78±0.03	1.657	0.109	1.713	0.098	0.409	0.686
R-SLFP	0.82±0.06	0.80±0.03	0.79±0.04	1.702	0.100	0.184	0.855	1.612	0.119
L-SLFT	0.84±0.07	0.81±0.04	0.79±0.03	2.599	0.015	1.702	0.100	1.328	0.196
R-SLFT	0.83±0.07	0.80±0.04	0.78±0.04	2.200	0.037	1.662	0.108	1.081	0.290
L-UNC	0.89±0.06	0.86±0.05	0.82±0.03	**3.888**	**0.001**	2.785	0.010	1.322	0.198
R-UNC	0.89±0.06	0.87±0.04	0.84±0.02	**3.579**	**0.001**	2.750	0.011	1.191	0.244

Bold characters indicate statistically significant results after t-test for indipendent samples and Bonferroni's correction (p threshold 0.05/18=0.003). MD values ($x10^{-3}mm^2/s$) are expressed as mean±standard deviation.

Abbreviations: AD=Alzheimer's Disease; DLB=dementia with Lewy bodies; ATR=anterior thalamic radiation, CAB=cingulum-angular (infracallosal) bundle, CCG=cingulum-cingulate gyrus (supracallosal) bundle, CTS=corticospinal tract, ILF=inferior longitudinal fasciculus, SLFP=superior longitudinal fasciculus-parietal bundle, SLFT=superior longitudinal fasciculus-temporal bundle, UNC=uncinate fasciculus, FMAJ=corpus callosum-forceps major, FMIN=corpus callosum-forceps minor.

Supplementary table 3. Radial diffusivity (RD) values for left and right white matter tracts.

Tract	DLB	AD	Controls	DLB vs.		AD vs.		DLB vs. AD	
				t_{27}	p	t_{27}	p	t_{26}	p
FMAJ	0.70±0.18	0.68±0.17	0.58±0.09	2.425	0.022	2.227	0.034	0.308	0.761
FMIN	0.71±0.10	0.70±0.12	0.68±0.09	0.684	0.500	0.407	0.688	0.215	0.832
L-ATR	0.66±0.05	0.64±0.06	0.62±0.02	2.316	0.028	1.247	0.223	0.519	0.608
R-ATR	0.67±0.04	0.65±0.05	0.63±0.02	2.979	0.006	1.647	0.111	0.856	0.400
L-CAB	0.73±0.07	0.79±0.08	0.70±0.04	1.267	0.216	**4.022**	**0.000**	-2.445	0.022
R-CAB	0.71±0.06	0.77±0.10	0.67±0.07	1.719	0.097	**3.445**	**0.002**	-2.182	0.038
L-CCG	0.59±0.05	0.58±0.06	0.53±0.03	**3.763**	**0.001**	2.554	0.017	0.556	0.583
R-CCG	0.61±0.07	0.60±0.04	0.55±0.05	2.655	0.013	3.121	0.004	0.381	0.706
L-CST	0.57±0.09	0.53±0.05	0.51±0.03	2.279	0.031	1.295	0.206	1.385	0.178
R-CST	0.57±0.07	0.56±0.07	0.53±0.03	2.091	0.046	1.674	0.106	0.244	0.809
L-ILF	0.68±0.07	0.68±0.06	0.63±0.04	2.593	0.015	2.639	0.014	0.064	0.949
R-ILF	0.68±0.08	0.68±0.06	0.63±0.03	2.446	0.021	**3.264**	**0.003**	-0.023	0.982
L-SLFP	0.65±0.08	0.63±0.05	0.60±0.04	1.867	0.073	1.599	0.121	0.633	0.532
R-SLFP	0.66±0.06	0.63±0.03	0.63±0.04	1.651	0.110	0.403	0.690	1.391	0.176
L-SLFT	0.65±0.07	0.61±0.04	0.60±0.04	2.594	0.015	0.904	0.374	1.857	0.075
R-SLFT	0.65±0.07	0.62±0.04	0.60±0.04	2.055	0.050	1.168	0.253	1.287	0.210
L-UNC	0.72±0.07	0.69±0.05	0.64±0.02	**3.754**	**0.001**	**3.431**	**0.002**	1.035	0.310
R-UNC	0.71±0.06	0.70±0.04	0.66±0.01	**3.715**	**0.001**	**3.544**	**0.001**	0.732	0.471

Bold characters indicate statistically significant results after t-test for indipendent samples and Bonferroni's correction (p threshold 0.05/18=0.003). RD values ($x10^{-3}mm^2/s$) are expressed as mean±standard deviation.

Abbreviations: AD=Alzheimer's Disease; DLB=dementia with Lewy bodies; ATR=anterior thalamic radiation, CAB=cingulum-angular (infracallosal) bundle, CCG=cingulum-cingulate gyrus (supracallosal) bundle, CTS=corticospinal tract, ILF=inferior longitudinal fasciculus, SLFP=superior longitudinal fasciculus-parietal bundle, SLFT=superior longitudinal fasciculus-temporal bundle, UNC=uncinate fasciculus, FMAJ=corpus callosum - forceps major, FMIN=corpus callosum-forceps minor.

Supplementary table 4. Axial diffusivity (DA) values for left and right white matter tracts.

Tract	DLB	AD	Controls	DLB vs.		AD vs.		DLB vs. AD	
				t_{27}	p	t_{27}	t_{27}	p	t_{27}
FMAJ	1.57±0.14	1.55±0.11	1.51±0.06	1.453	0.158	1.337	0.193	0.288	0.775
FMIN	1.43±0.10	1.40±0.07	1.39±0.07	0.988	0.332	0.199	0.844	0.827	0.416
L-ATR	1.20±0.06	1.18±0.05	1.14±0.04	2.809	0.009	2.254	0.032	0.844	0.407
R-ATR	1.20±0.05	1.18±0.05	1.14±0.04	**3.835**	**0.001**	2.795	0.009	1.162	0.256
L-CAB	1.19±0.06	1.24±0.08	1.12±0.06	2.985	0.006	**4.557**	**0.000**	-2.073	0.048
R-CAB	1.16±0.04	1.22±0.12	1.11±0.07	2.341	0.027	3.063	0.005	-1.756	0.091
L-CCG	1.28±0.07	1.25±0.06	1.24±0.07	1.336	0.193	0.481	0.635	0.954	0.349
R-CCG	1.22±0.05	1.23±0.12	1.16±0.06	2.831	0.009	2.045	0.051	-0.316	0.754
L-CST	1.25±0.06	1.24±0.05	1.21±0.05	1.947	0.062	1.719	0.097	0.477	0.637
R-CST	1.24±0.06	1.23±0.09	1.20±0.05	2.098	0.045	1.341	0.191	0.272	0.788
L-ILF	1.33±0.07	1.32±0.07	1.28±0.06	2.222	0.035	1.893	0.069	0.310	0.759
R-ILF	1.28±0.08	1.27±0.09	1.26±0.05	0.592	0.558	0.427	0.673	0.088	0.930
L-SLFP	1.14±0.08	1.15±0.06	1.12±0.04	1.148	0.261	1.727	0.096	-0.107	0.915
R-SLFP	1.16±0.06	1.12±0.04	1.13±0.04	1.618	0.117	-0.202	0.841	1.822	0.080
L-SLFT	1.21±0.07	1.20±0.04	1.17±0.04	2.032	0.052	2.686	0.012	0.032	0.975
R-SLFT	1.18±0.08	1.17±0.05	1.13±0.03	2.360	0.026	2.287	0.030	0.599	0.554
L-UNC	1.24±0.05	1.21±0.05	1.18±0.04	**3.464**	**0.002**	1.341	0.191	1.856	0.075
R-UNC	1.26±0.07	1.22±0.05	1.20±0.04	2.988	0.006	1.238	0.226	1.770	0.088

Bold characters indicate statistically significant results after t-test for indipendent samples and Bonferroni's correction (p threshold 0.05/18=0.003). DA values ($x10^{-3}mm^2/s$) are expressed as mean±standard deviation.

Abbreviations: AD=Alzheimer's Disease; DLB=dementia with Lewy bodies; ATR=anterior thalamic radiation, CAB=cingulum-angular (infracallosal) bundle, CCG=cingulum-cingulate gyrus (supracallosal) bundle, CTS=corticospinal tract, ILF=inferior longitudinal fasciculus, SLFP=superior longitudinal fasciculus-parietal bundle, SLFT=superior longitudinal fasciculus-temporal bundle, UNC=uncinate fasciculus, FMAJ=corpus callosum-forceps major, FMIN=corpus callosum-forceps minor.

Supplementary Table 5. Effect of deep white matter hyperintensity (DHWM) on DTI changes among groups.

Metric	Tract	F	p
FA	R-ILF	0.749	0.392
MD	L-CAB	0.079	0.781
	R-CAB	0.044	0.835
	L-CCG	0.068	0.796
	L-UNC	1.128	0.295
	R-UNC	0.378	0.542
RD	L-CAB	0.277	0.601
	R-CAB	0.199	0.658
	R-CCG	0.029	0.866
	R-ILF	1.884	0.178
	L-UNC	2.172	0.149
	R-UNC	0.921	0.343
DA	R-ATR	3.256	0.079
	L-CAB	0.045	0.833
	R-CAB	0.089	0.767
	L-UNC	0.000	1.000

Statistical outcomes derived from ANCOVA.

Abbreviations: FA=fractional anisotropy; MD=mean diffusivity; RD=radial diffusivity; DA=axial diffusivity; ATR=anterior thalamic radiation, CAB=cingulum-angular (infracallosal) bundle, CCG=cingulum-cingulate gyrus (supracallosal) bundle, ILF=inferior longitudinal fasciculus, UNC=uncinate fasciculus.

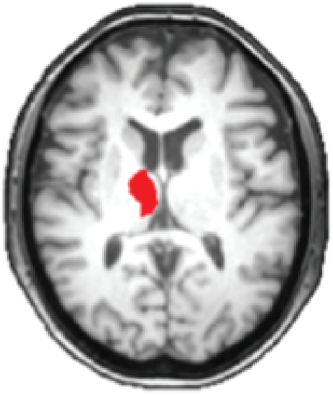

Supplementary figure. Representative image shows the right medio-dorsal region of thalamic which projects to frontal cortex. The thalamic region is colored in red and overlapped on T_1 structural image.
